# Efficacy of reduced-intensity or no heparin versus standard heparin anticoagulation in patients on extracorporeal membrane oxygenation: a systematic review and meta-analysis

**DOI:** 10.3389/fmed.2026.1767978

**Published:** 2026-01-29

**Authors:** Tianyu Zhang, Lingling Cheng, Zhen Cheng, Aili Shi, Weiying Shao

**Affiliations:** 1 School of Public Health and Nursing, Hangzhou Normal University, Hangzhou, China; 2 Zhejiang Provincial People’s Hospital, Hangzhou, China; 3 Tongde Hospital of Zhejiang Province, Hangzhou, China; 4 People’s Hospital of Quzhou, Quzhou, China

**Keywords:** anticoagulation, critical care, extracorporeal membrane oxygenation, management, meta-analysis

## Abstract

**Objective:**

This study aims to evaluate the efficacy of reduced-intensity or no heparin anticoagulation strategy in comparison to standard anticoagulation strategy during extracorporeal membrane oxygenation (ECMO) support.

**Materials and methods:**

Systematic literature review and meta-analysis, complying with the PRISMA guidelines (PROSPERO-CRD42025633878).

**Results:**

Eleven studies comprising 958 patients were included in the analysis. Four studies included only patients treated with veno-venous extracorporeal membrane oxygenation (V-V ECMO) for acute respiratory distress syndrome or respiratory failure, two studies focused exclusively on patients treated with veno-arterial extracorporeal membrane oxygenation (V-A ECMO), and five studies included a mixture of patients with both modalities. Most studies (*n* = 8) were of high quality, as indicated by a Newcastle-Ottawa Scale score of ≥ 6. The overall incidence of bleeding complications was 34% (95% confidence interval (CI): 0.35–0.67), without heterogeneity observed among the studies (*I*^2^ = 43%). The overall incidence of thrombotic events was 14.6% (95% CI: 0.65–1.54; *I*^2^ = 49%). The overall in-hospital mortality was 49% (95% CI: 0.67–1.21; *I*^2^ = 41%), while the red blood cell transfusion rate was 41.2% (95% CI: 0.08–1.02; *I*^2^ = 76%).

**Conclusion:**

Reduced-intensity or no heparin anticoagulation appears to be a feasible and safe strategy, demonstrating the potential to reduce bleeding complications without a significant increase in thrombotic events, and may be associated with improved patient outcomes.

**Systematic review registration:**

https://www.crd.york.ac.uk/PROSPERO/view/CRD42025633878, identifier CRD42025633878.

## 1 Introduction

Extracorporeal membrane oxygenation (ECMO), an advanced form of extracorporeal life support, involves drawing blood from the vein using a blood pump, oxygenating it, and then reinfusing it into the body. It is primarily utilized as a continuous cardiopulmonary replacement support therapy (1). Introduced into clinical practice at the end of the last century, ECMO has gradually gained prominence and is now regarded as one of the important life support modalities in critical care medicine (2–4). It has been widely employed in patients with fulminant myocarditis, cardiogenic shock, cardiopulmonary failure, and other conditions (5). However, research has documented that ECMO use can lead to specific complications. This is not a matter of speculation; rather, these adverse events are well-documented in the clinical research (6, 7), among which bleeding complications, including intracranial and gastrointestinal hemorrhages, are particularly common and severe, accounting for about 27–60% of all complications (5, 8).

The occurrence of complications is strongly associated with anticoagulant therapy (9, 10). The Extracorporeal Life Support Organization (ELSO) has issued guidelines for anticoagulation during ECMO, recommending the maintenance of the activated clotting time (ACT) and the activated partial thromboplastin time (aPTT) between 180–220 and 50–70 s, respectively. While the ACT and aPTT remain the most widely adopted and guideline-recommended parameters, alternative monitoring tools such as anti-Xa assay and viscoelastic tests are increasingly used in clinical practice (11). The present analysis included studies utilizing these various monitoring strategies; however, for the purpose of comparing anticoagulation intensity (low/no vs. standard), the outcomes were synthesized across studies irrespective of the specific monitoring tool used. Simultaneously, ELSO highlighted the challenges in fully implementing anticoagulation therapy in accordance with the recommended guidelines due to the varying conditions of individual patients. Therefore, various medical centers have explored different anticoagulation strategies for ECMO. Some centers have implemented veno-arterial extracorporeal membrane oxygenation (V-A ECMO) without anticoagulation in patients with significant bleeding risk following cardiac surgery and cardiopulmonary bypass (12, 13). Additionally, some studies have indicated that low-intensity anticoagulation or the absence of systemic anticoagulation may reduce the incidence of bleeding and/or thrombotic events and some researchers have also published their anticoagulation experiences and strategies in this context (14–16). However, it remains controversial whether lower-intensity anticoagulation or no systemic anticoagulation is truly superior to standard anticoagulation.

Therefore, we conducted a meta-analysis to compare bleeding complications, thrombotic events, in-hospital mortality, and transfusion requirement events in patients with ECMO receiving different anticoagulation strategies.

## 2 Materials and methods

Meta-analysis was conducted in accordance with Preferred Reporting Items for Systematic Reviews and Meta-Analyses (PRISMA) guidelines for randomized controlled trials (RCTs) and the Meta-analysis of Observational Studies in Epidemiology (MOOSE) reporting guidelines for observational studies. The meta-analysis, registered with the International Prospective Register of Systematic Reviews (PROSPERO) (CRD42025633878) and published online, provides comprehensive details, including literature search strategy, the purpose of meta-analysis, literature selection, and inclusion-exclusion criteria, data collection entries, and quality assessment of included studies.

### 2.1 Search strategy and literature selection

Eligible studies were identified by consulting the Cochrane Library, PubMed, EMBASE, and Web of Science, without date or language restrictions. Keywords and MeSH terms relevant to the exposure of interest were utilized in relevant combinations: “ECMO,” “ECLS,” “extracorporeal life support,” “extracorporeal membrane oxygenation,” “low-dose anticoagulation,” “low anticoagulation,” “restrict anticoagulation,” “low-dose,” “low dose,” “standard anticoagulation,” “therapeutic anticoagulation,” and “systemic anticoagulation.” The literature search covered studies from the inception of each database up to January 2025. In addition, trial registries were searched, and reference lists were traced for studies. The detailed search protocol is provided in Supplementary Figure 1.

The inclusion criteria were established based on the “PICOS” principle: (1) Study population (P): Adult patients treated with ECMO; (2) intervention (I): Low-intensity or no anticoagulation; (3) comparator (C): Standard anticoagulation or high-intensity anticoagulation strategies; (4) outcomes (O): Incidence of bleeding and thrombotic events, in-hospital mortality, and red blood cell transfusion events; (5) study design (S): RCTs, prospective or retrospective observational cohort studies. Articles were excluded based on the following criteria: (1) Review, case reports, and conference abstracts; (2) incomplete outcome indicators; (3) lack of a specific study protocol; (4) inability to obtain the full text.

### 2.2 Data extraction

Two researchers screened the titles and abstracts of the identified literature based on the inclusion criteria. For studies meeting the inclusion criteria, the full texts were thoroughly reviewed. In case of disagreement, the two researchers engaged in discussion or consulted the third researcher to determine the final included literature. The following information was extracted from the literature that met the inclusion criteria: Author, publication year, country, study type and design, sample size, baseline data of patients, interventions, bleeding and thrombotic complications, in-hospital mortality, and other outcome indicators.

### 2.3 Study outcomes

The primary outcome was the incidence of bleeding complications, while the secondary outcomes included thrombotic events, in-hospital mortality, and transfusion requirement. And the outcome of “transfusion requirement” was defined as the incidence of packed red blood cell (RBC) transfusion. Transfusions of other blood products, like plasma were not included in this outcome measure.

### 2.4 Statistical analysis

RevMan (version 5.4) software was used to conduct meta-analysis. For continuous variables, the mean difference (MD) was employed as the effect analysis statistic, while for dichotomous variables, the risk ratio (RR) or odds ratio (OR) was used as the effect analysis statistic. The X^2^ test and I^2^ statistic were used to evaluate the heterogeneity among the studies (*P* > 0.1 and *I*^2^ < 50%) using a fixed effect model. In cases of significant heterogeneity (*P* ≤ 0.1 and *I*^2^ ≥ 50%), the random effect model was employed for analysis.

## 3 Results

### 3.1 Results of study selection

**FIGURE 1:**
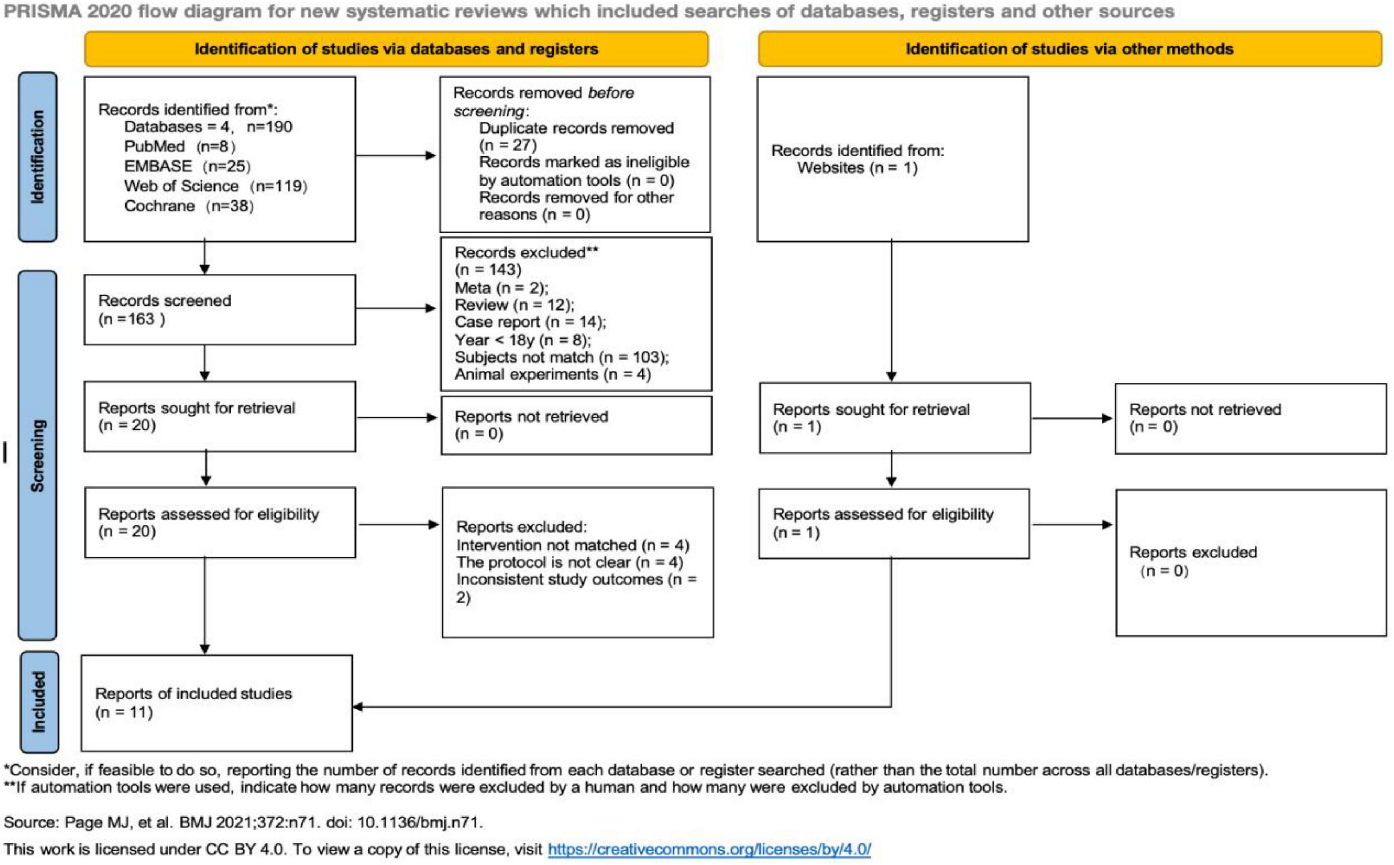
Flow chart for selecting the articles in this meta-analysis.

Figure 1 illustrates the literature inclusion process. A total of 190 articles were obtained through the search, and one additional relevant article was supplemented through other sources, bringing the total to 191 articles. After excluding 27 duplicate articles, 164 articles remained, and 11 articles were included in the final meta-analysis following layer-by-layer screening (17–27).

### 3.2 Characteristics and quality assessment of included studies

The basic characteristics of the included studies are demonstrated in Table 1. One study (21) was an RCT, while the remaining 10 (17–20, 22–27) were retrospective cohort studies, with 3 studies (21, 24, 26) using aPTT for monitoring, 3 studies using ACT for monitoring (19, 20, 25), and the remaining studies employing mixed indicators for monitoring. A total of 958 patients were enrolled, with 167 patients receiving no anticoagulation, 404 receiving low anticoagulation, and 387 receiving standard anticoagulation. Regarding the anticoagulation strategies, among the 11 included studies, none explicitly described a protocol involving planned switching from one anticoagulation method to another during the ECMO course. Similarly, the protocols for the reversal of anticoagulation or the management of anticoagulation cessation were not detailed in the included literatures. Comparisons in this study were based on the intensity of anticoagulation initially prescribed in each study (low/no heparin vs. Standard heparin). One study (21) reported the method of generating the randomized sequence, although double-blinding was not possible due to the intervention measures and other reasons. However, as the outcome indicators of this study were objective, the absence of blinding had little impact on the results. Randomization could not be performed in the remaining studies as they were retrospective. Additionally, this study analyzed indicators with great heterogeneity in the research results and pointed out the sources.

**TABLE 1:** Characteristics of the included studies.

Studies	Country	Study types	Number of patients		Anticoagulation target		Outcomes
			Low/Free	Standard	Low/No	Standard	
Robinson et al. (17)	USA	RCS	14	13	Anti-Xa 0.3–0.5 units/mL	Anti-Xa 0.3–0.7 units/mL	① ② ③
Seeliger et al. (18)	GER	RCS	101	117	aPTT 35–40 s	ACT 140–180 s	① ② ③ ④
Hong et al. (19)	KOR	RCS	14	29	ACT < 150 s	ACT 180–200 s	① ② ③
Carter et al. (20)	USA	RCS	23	17	ACT 140–180 s	ACT 180–200 s	① ② ③
Aubron et al. (21)	AUS	RCT	16	16	aPTT < 45 s	aPTT 50–70 s	① ② ③ ④
Wood et al. (22)	USA	RCS	131	72	NA	ACT 180–220 s/aPTT 54–71 s	① ② ③ ④
Kurihara et al. (23)	USA	RCS	36	38	NA	ACT 160–180 s/aPTT 50–70 s	① ④
Krueger et al. (24)	GER	RCS	61	NA	NA	NA	① ② ③
Raman et al. (25)	USA	RCS	52	50	NA	ACT 180–220 s	① ② ③
Hu et al. (26)	CHN	RCS	53	35	aPTT < 50 s	aPTT ≥ 50 s	① ② ③ ④
Zhao et al. (27)	CHN	RCS	70	NA	Heparin 3,000 Units	NA	② ③

RCS, retrospective cohort study; RCT, randomized controlled trial; ACT, activated clotting time; Aptt, activated partial thromboplastin time. ① Bleeding complication; ② Thrombotic events; ③ In-hospital mortality; ④ Red blood cell transfusion.

The quality of RCTs was evaluated using the Cochrane Risk of Bias Assessment tool (28). The evaluation criteria included randomized allocation method, allocation concealment, blinding, completeness of data results, selective reporting of research results, and other sources of bias. Each item was evaluated as “low risk of bias,” “high risk of bias,” or “unclear.” The quality of cohort studies was evaluated using the Newcastle-Ottawa Scale (NOS) (29), which included 8 items across 3 dimensions: Selection of study subjects, comparability between groups, and outcome measurement. The full score was 9 points, where a score of 0–4 points indicated low quality, 5–6 points indicated moderate quality, and a score of ≥ 7 points indicated high quality. One RCT study was included in this study, and the quality evaluation results are presented in Supplementary Figure 2. A total of 10 cohort studies were included, and their methodological quality is evaluated in Supplementary Table 1. Among these, 2 studies are of low quality, 4 are of medium quality, and 4 are of high quality. The results are detailed in Table 1 and Supplementary Figure 2.

### 3.3 Results of meta-analysis

#### 3.3.1 Effect of low/no heparin anticoagulation on the incidence of bleeding complications and thrombotic events in patients with ECMO

**FIGURE 2:**
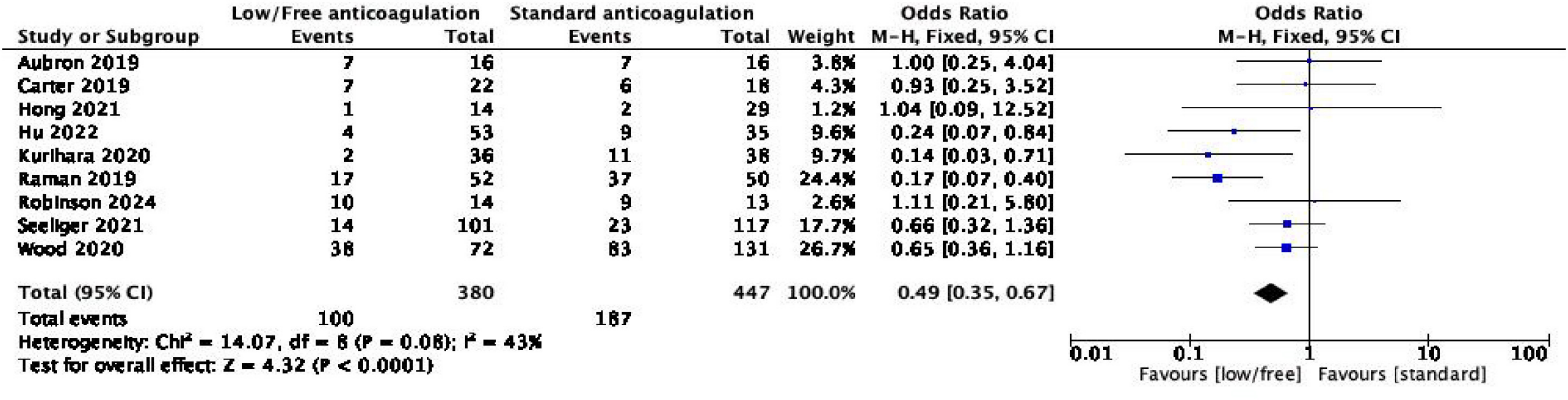
Forest plot of the meta-analysis of bleeding complications.

Of the 11 included studies, 9 (17–23, 25, 26) reported data on the incidence of bleeding complications and were thus included in this meta-analysis. The data from these studies were pooled for analysis and further examination. The pooled results demonstrated low heterogeneity among the studies (*I*^2^ = 43%, *P* = 0.08), and thus the fixed-effect model was employed for analysis. The findings revealed that low/no heparin anticoagulation could reduce the incidence of bleeding complications in patients with ECMO [OR = 0.49, 95% confidence interval (CI) (0.35, 0.67), and *P* < 0.0001]. Additionally, the meta-analysis demonstrated that the incidence of intracranial and gastrointestinal tract hemorrhage was lower in the low/no heparin anticoagulation group compared to the standard anticoagulation group, without heterogeneity between studies (*I*^2^ = 0), and the results were statistically significant. The study results are detailed in Figure 2.

**FIGURE 3:**
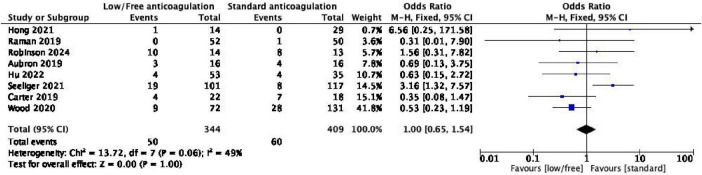
Forest plot of the meta-analysis of thrombotic events.

Eight of the nine studies reporting bleeding complications (17–22, 25, 26) also reported data on thrombotic events. After combining the data, the heterogeneity among the studies was found to be low (*I*^2^ = 49%, *P* = 0.06); accordingly, the fixed effect model was used for meta-analysis. The results demonstrated that there was no statistically significant difference in the incidence of thrombotic events between the low/no heparin anticoagulation group and the standard anticoagulation group [OR = 1.00, 95% CI (0.65, 1.54), and *P* = 1.00]. The confidence interval indicates that the data are compatible with both a potential reduction and a potential increase in thrombotic risk associated with low/no heparin anticoagulation. The detailed results are presented in Figure 3.

#### 3.3.2 Effect of low/no heparin anticoagulation on in-hospital mortality

**FIGURE 4:**
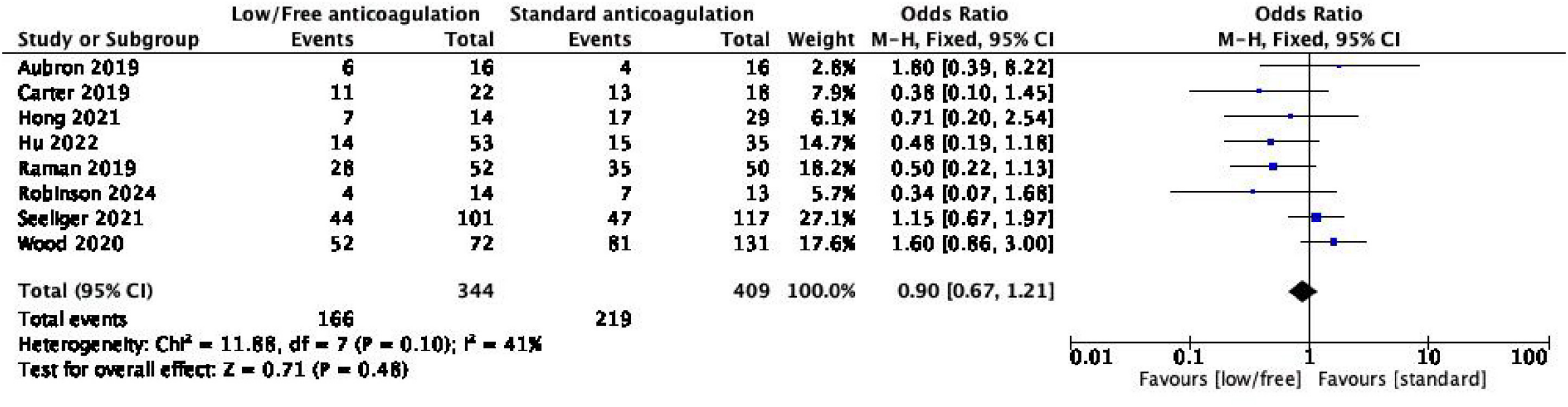
Forest plot of the meta-analysis of in-hospital mortality.

Eight studies (17–22, 25, 26) provided mortality data, and there was low heterogeneity among the studies after combined analysis (*I*^2^ = 41%, *P* = 0.10); thus, the fixed effect model was used for analysis. The results demonstrated that there was no significant difference in mortality between the low anticoagulation group and the standard anticoagulation group, indicating that low anticoagulation intensity did not increase the mortality of patients with ECMO [OR = 0.90, 95% CI (0.67, 1.21)]. The results are detailed in Figure 4.

#### 3.3.3 Effect of anticoagulation intensity on red blood cell transfusion

**FIGURE 5:**
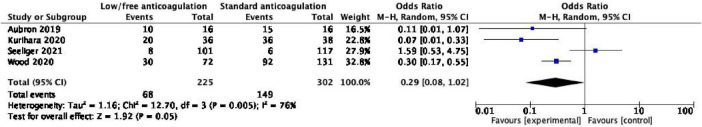
Forest plot of the meta-analysis of red blood cell transfusion events.

Of the 11 studies (17–27) included in the analysis, 4 (18, 21–23) reported data on transfusion requirement, involving 68 patients in the low/no heparin anticoagulation group and 149 in the standard anticoagulation group. The results of the meta-analysis demonstrated no significant difference in transfusion requirement between the two groups [OR = 0.29, 95% CI (0.08, 1.02)]. However, the results of the combined analysis revealed a significant heterogeneity among the studies (*I*^2^ = 76%, *P* = 0.005). To identify the source of heterogeneity, a sensitivity analysis was conducted by excluding studies one by one. After excluding the study by Seeliger et al. (18), the heterogeneity (*I*^2^ = 43%, *P* = 0.17) was significantly reduced. This reduction suggested that the anticoagulation strategy and transfusion threshold adopted by Seeliger et al. (18) differed considerably from other studies, resulting in greater heterogeneity. The results are detailed in Figure 5.

## 4 Discussion

Anticoagulation management during ECMO remains highly variable across centers, with no universally accepted standard. Protti et al. (30) highlighted this heterogeneity in a 2020 worldwide survey of 273 anticoagulation strategies, underscoring the challenge of balancing thrombotic and hemorrhagic risks during ECMO support. In response, some centers have adopted lower anticoagulation targets, though whether such approaches improve clinical outcomes compared with conventional intensity regimens remains uncertain. This meta-analysis aimed to compare the safety and efficacy of low-intensity versus standard-intensity anticoagulation in ECMO patients. Our principal findings are as follows: first, low-intensity anticoagulation was associated with a reduction in bleeding complications; second, no significant differences were observed in thrombotic events or in-hospital mortality between strategies; and third, there was no statistically significant difference in transfusion requirements, though a non-significant trend favoring liberal anticoagulation was noted.

The reduced incidence of bleeding with low-intensity anticoagulation aligns with physiological expectations and previous clinical observations. Although the ELSO guidelines recommend targeting an ACT of 180–220 s and aPTT of 50–70 s, these ranges are largely empiric (31). Multiple institutions now employ targets below these values, and some even adopt minimally anticoagulated or anticoagulation-free strategies, particularly in high-bleeding-risk scenarios (19–22). A recent RCT in adults on V-V ECMO (32) reported major bleeding in 7.1% of patients under low-intensity anticoagulation versus 14.3% in those receiving moderate-intensity therapy, supporting the safety of lower targets. Similarly, in V-A ECMO, Descamps et al. (15) identified anti-Xa > 0.46 IU/mL as an independent risk factor for bleeding, reinforcing the potential benefit of reduced anticoagulation intensity. Krueger et al. (24) further demonstrated that prophylactic-dose anticoagulation did not increase mortality. Technological advances in circuit biocompatibility and individualized monitoring—such as anti-Xa-guided dosing (33)—may facilitate safer implementation of low-intensity protocols, particularly in patients with contraindications to full systemic anticoagulation (34, 35). It is noteworthy that an increasing number of centers are shifting from traditional tests like ACT to anti-Xa activity for routine monitoring. This evolution in practice is driven by evidence suggesting that ACT and aPTT correlate weakly with heparin dose and are susceptible to interference from multiple factors, coagulation factor deficiencies, potentially leading to inaccurate assessment of anticoagulation intensity in ECMO patients. In contrast, anti-Xa monitoring offers a more stable and direct measure of heparin effect, which may improve dosing precision and reduce the frequency of dose adjustments (11, 36, 37). Consequently, this evolving practice underscores the importance of focusing on the achieved anticoagulant intensity rather than the specific monitoring tool itself when interpreting and comparing the outcomes of studies on different anticoagulation strategies.

Notably, we found no significant increase in thrombotic events with low-intensity anticoagulation. Thrombosis during ECMO is multifactorial, influenced by circuit surface interactions, flow dynamics, and patient-specific coagulopathy (31). However, conventional monitoring primarily detects macroscopic circuit thrombosis; subclinical microthrombi may go unrecognized. Only two included studies (20, 23) stratified thrombotic events by type and location, limiting pathophysiological insight. While some reports suggest anticoagulation-free ECMO is feasible in selected cases (24), the risk of occult thrombosis under very low anticoagulation remains poorly characterized. Future studies incorporating viscoelastic testing or advanced imaging may improve detection and illuminate this unresolved issue.

In-hospital mortality did not differ significantly between anticoagulation strategies. This may reflect counterbalancing effects: while bleeding complications are reduced with lower anticoagulation intensity, thrombotic risk is not fully eliminated. Both major bleeding and thrombosis have been independently associated with mortality (7, 38), and their competing risks might neutralize the net effect of anticoagulation intensity on survival. Notably, Nunez et al. (39) reported that frequent anticoagulation interruptions due to bleeding complicate management and underscore the need for highly individualized therapy. The lack of detailed reporting on bleeding subtypes (e.g., intracranial vs. gastrointestinal) and their respective contributions to mortality precluded further subgroup analysis—an important limitation that future studies should address.

No significant difference in transfusion requirements was observed between groups, despite a trend toward reduced transfusion in the liberal anticoagulation group (OR = 0.29). The substantial heterogeneity (*I*^2^ = 76%) initially detected was largely attributable to variations in transfusion protocols, particularly the restrictive strategy employed by Seeliger et al. (20). After excluding this study, heterogeneity dropped to moderate levels (*I*^2^ = 43%), yet the result remained non-significant. Transfusion practices in ECMO patients are influenced by institutional protocols, severity of illness, and physiological thresholds, with wide variation in recommended hemoglobin targets (40–42). Coagulopathy during ECMO is multifactorial, involving hemodilution, platelet dysfunction, inflammation, and iatrogenic factors (43, 44), which may dilute the isolated effect of anticoagulation intensity on transfusion needs.

Several limitations should be acknowledged. The included studies were predominantly observational and varied in anticoagulation targets, monitoring methods, and definitions of bleeding and thrombosis. The lack of patient-level data precluded adjustment for confounding factors, and clinical heterogeneity in ECMO modes and indications may have influenced outcomes. Second, we could not account for the potential confounding effects of concomitant medications (e.g., antiplatelet agents or anticoagulant drugs for other indications) that may independently influence the risks of bleeding and thrombosis. This lack of data represents an important source of bias that should be considered when interpreting the net effect of anticoagulation intensity alone. Furthermore, the possibility of undetected microthrombotic events under low-intensity anticoagulation warrants cautious interpretation.

## 5 Conclusion

In conclusion, this meta-analysis suggests that low-intensity anticoagulation during ECMO reduces bleeding complications without significantly increasing thrombotic events or mortality. However, no firm conclusions can be drawn regarding transfusion requirements due to significant heterogeneity and limited data. These findings support the individualization of anticoagulation therapy based on patient-specific risks and real-time hemostatic monitoring. Future large-scale, randomized trials are needed to validate these results and to identify optimal anticoagulation targets for distinct ECMO populations.

## Data Availability

The original contributions presented in this study are included in this article/Supplementary material, further inquiries can be directed to the corresponding authors.
