# Supplementary material for: Efficacy of reduced-intensity or no heparin versus standard heparin anticoagulation in patients on extracorporeal membrane oxygenation: a systematic review and meta-analysis
Source: Front Med (Lausanne). 2026 Jan 29;13:1767978. doi: 10.3389/fmed.2026.1767978 (PMC12894395; doi:10.3389/fmed.2026.1767978)
Supplement: Supplementary file 1 [file Table_1.docx]

**Supplementary Material**

#1 "Extracorporeal Membrane Oxygenation"[MeSH Terms]

#2 "extracorporeal membrane oxygenation"[Title/Abstract] OR "ECMO"[Title/Abstract] OR "extracorporeal life support"[Title/Abstract] OR "ECLS"[Title/Abstract]

#3 #1 OR #2

#4 "low-dose anticoagulation"[Title/Abstract] OR "low anticoagulation"[Title/Abstract] OR "low-dose"[Title/Abstract] OR "low dose"[Title/Abstract] OR "restrict anticoagulation"[Title/Abstract] OR "standard anticoagulation"[Title/Abstract] OR "therapeutic anticoagulation"[Title/Abstract] OR "systemic anticoagulation"[Title/Abstract]

#5 #3 AND #4

Figure 1. Search protocol using PubMed as an example


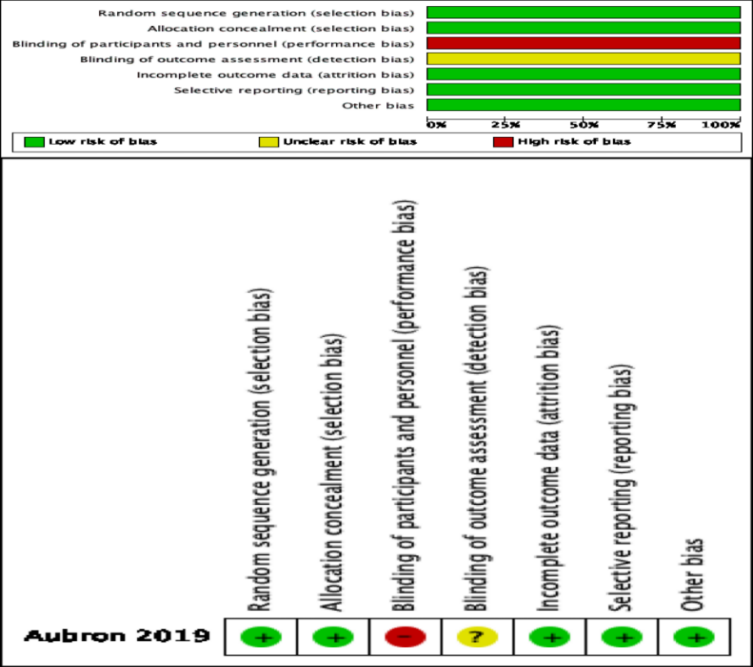
Figure 2. Methodological quality assessment for the included RCT (n=1)

Table 1. Methodological quality assessment for the included cohort studies (n=10)

| Study (Author, Year) | Selection | Comparability | Outcome | Total |
| --- | --- | --- | --- | --- |
| *Robinson, 2024^[13]^* | 4 | 1 | 1 | 6 |
| *Seeliger, 2021^[14]^* | 4 | 2 | 1 | 7 |
| *Hong, 2021^[15]^* | 4 | 2 | 1 | 7 |
| *Carter, 2019^[16]^* | 4 | 1 | 1 | 6 |
| *Wood, 2020^[18]^* | 4 | 1 | 1 | 6 |
| *Kurihara, 2020^[19]^* | 4 | 2 | 1 | 7 |
| *Krueger, 2017^[20]^* | 3 | 0 | 1 | 4 |
| *Raman, 2019^[21]^* | 4 | 0 | 3 | 7 |
| *Hu, 2022^[22]^* | 4 | 1 | 1 | 6 |
| Zhao, 2022*^[23]^* | 2 | 0 | 1 | 3 |
